# Stress and drug resistance in cancer

**DOI:** 10.20517/cdr.2019.016

**Published:** 2019-09-19

**Authors:** Renée L. Flaherty, Marta Falcinelli, Melanie S. Flint

**Affiliations:** School of Pharmacy and Biomolecular Sciences, University of Brighton, Moulsecoomb, Brighton, BN2 4GJ, UK.

**Keywords:** Cancer, catecholamines, dexamethasone, drug resistance, glucocorticoids

## Abstract

Patients diagnosed with cancer often undergo considerable psychological distress, and the induction of the psychological stress response has been linked with a poor response to chemotherapy. The psychological stress response is mediated by fluctuations of the hormones glucocorticoids (GCs) and catecholamines. Binding to their respective receptors, GCs and the catecholamines adrenaline/noradrenaline are responsible for signalling a wide range of processes involved in cell survival, cell cycle and immune function. Synthetic GCs are also often prescribed as co-medication alongside chemotherapy, and increasing evidence suggests that GCs may induce chemoresistance in multiple cancer types. In this review, we bring together evidence linking psychological stress hormone signalling with resistance to chemo- and immune therapies, as well as mechanistic evidence regarding the effects of exogenous stress hormones on the efficacy of chemotherapies.

## Introduction

The psychological stress response is designed to facilitate a response to perceived threat in order to maintain homeostatic equilibria. In humans, the stress response is controlled by fluctuations in hormonal secretions, primarily glucocorticoids and catecholamines, under the control of the hypothalamic-pituitary-adrenal (HPA) axis and sympathetic nervous system (SNS)^[1]^. Activation of these systems results in host priming responses including vasoconstriction and tachycardia, as well as metabolic catabolism and redirection of resources to stressed sites, designed to allow for a “fight-or-flight” response^[2]^. However, in the recent evolution of humans, what promotes the initiation of the stress response has changed dramatically, while the actual stress response has not. Modern environmental stressors identified include socioeconomic burden, social isolation and negative life events as opposed to life-threatening survival situations. As such this presents an evolutional disparity between stimuli and response, where responses designed for a “fight-or-flight” such as an increase in appetite to fuel increase energy expenditure, are chronically overstimulated. This exposure to sustained stress hormone signalling has been linked to an increased risk of diseases such as hypertension, immune dysfunction and cancer^[3]^. The main objective of the present review is to describe the available knowledge linking psychological stress hormone signalling with resistance to chemo- and immune therapies in human and mouse models. A secondary goal is to discuss the mechanistic evidence regarding the effects of exogenous stress hormones on the efficacy of chemotherapies.

## Glucocorticoids

**Figure 1:**
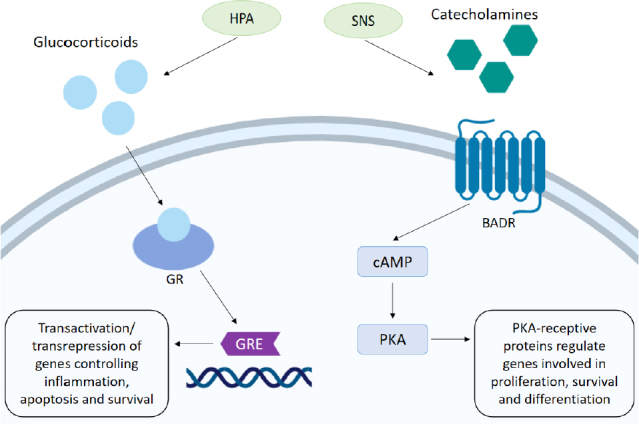
Stress hormone mechanism of action. The HPA signals the release of glucocorticoids. Activation of the GR mediates transactivation and transrepression of genes through binding to the GRE, which controls the expression of genes involved in inflammation, survival and apoptosis. Stimulation of the SNS promotes the release of catecholamines. Activation of the BADR by adrenaline/noradrenaline stimulates synthesis of cAMP which in turn activates PKA. Phosphorylation of a number of PKA-receptive proteins involved in cell survival, proliferation and gene transcription can then occur. HPA: hypothalamic-pituitary-axis; GR: glucocorticoid receptor; GRE: glucocorticoid response element; SNS: sympathetic nervous system; BADR: beta-adrenergic receptor; cAMP: cyclic adenosine monophosphate; PKA: protein kinase A

Glucocorticoids (GCs) are regulated though the HPA axis and function in many roles, including roles in the circadian rhythm as well as mediating adaptive responses under the stress system^[4]^. The glucocorticoid cortisol, an important hormone released under stressful conditions, is synthesised from cholesterol in the adrenal cortex. It is released when corticotropin-releasing hormone in the hypothalamus triggers the anterior pituitary to release adrenocorticotropic hormone, in response to complex stress signalling from neurons and somatic cells. This in turn stimulates the release of cortisol by the adrenal cortex. Cortisol can then act upon a number of systems to regulate homeostatic mechanisms by binding to its cytoplasmic glucocorticoid receptor (GR), present on nearly every cell in the body^[5]^. Activation of the GR translocation to the nucleus where gene activation and transcription can be negatively or positively modulated. The GR is able to bind to specific glucocorticoid response elements (GREs) in gene promotor regions of DNA, and facilitate biphasic transcriptional activation (transactivation). GRs are also able to interact with transcription factors such as AP-1 and NF-κB, inhibiting their transcriptional ability, thus supressing gene expression by a mechanism known as transrepression^[6]^. In this way GC through the GR can regulate a number of crucial genes controlling metabolism, survival, and apoptosis^[7]^. GCs can also suppress immune responses and activate anti-inflammatory pathways by downregulating expression of certain interleukins (ILs), thus decreasing immune cell numbers and activation. This is thought to be a protective mechanism, directing the body’s resources away from non-essential processes^[8]^. The role of GC as anti-inflammatories and their role in suppressing the immune system has been extensively reviewed elsewhere^[9]^. Synthetic corticosteroids are regularly administered to cancer patients alongside conventional chemotherapies to combat hypersensitivities and reduce inflammation, as well as acting as antiemetics to relieve nausea and vomiting^[10]^. In lymphoid cells, GC are pro-apoptotic and are regularly used as an anti-cancer therapy, with the potent synthetic GC dexamethasone used regularly with success. However, evidence suggests they may have opposing effects in some solid tumours, contributing to resistance to chemotherapy [Figure 1].

## Catecholamines

The catecholamines adrenaline (A) and noradrenaline (NA) are produced in the adrenal medulla by chromaffin cells and released into circulation when stressors activate the SNS. Neural fibres of the SNS are able to release these neurotransmitters into all the major organ systems within seconds, allowing for rapid physiological responses. Once released the effects of catecholamines are mediated by α- or β-adrenergic receptors (ARs), which exist in subtypes (α1-, α2-, β1-, β2- and β3-) and are distributed throughout tissues accordingly. α-adrenergic receptors primarily mediate vasoconstriction and contraction of smooth muscle and are present on vascular muscle. β-adrenergic receptors also regulate muscle contraction with β1-adrenergic receptors located on myocardial muscle acting to increase blood pressure and heart rate thus increasing blood flow to skeletal muscles. β2-adrenergic receptors present on bronchial smooth muscles facilitate muscle relaxation and vasodilation and also stimulate glucose metabolism^[11]^. Activation of β-adrenergic receptors stimulates synthesis of cyclic adenosine monophosphate (cAMP) which acts as an effector in a range of cellular processes. As a result of cAMP upregulation activation of protein kinase A (PKA) can occur, which in turn allows phosphorylation of a number of PKA-receptive proteins involved in cell survival, proliferation and gene transcription. Induction of gene transcription occurs as a result of PKA-induced phosphorylation of transcription factors responsible for the transcription of genes promoting stress response elements, such as cell differentiation and metabolism^[12]^ [Figure 1].

### Mechanisms of the stress response

Stress hormones mediate a wide range of biological processes in the cancer setting, and as such the effects of stress hormone signalling on chemotherapies is thought to be due to various mechanisms and dependent on the chemotherapeutic agent. The effects of stress hormone signalling on the efficacy of various anti-cancer treatments is reviewed below.

### Taxanes

Taxanes such as Paclitaxel and Docetaxel are widely used chemotherapeutic agents capable of disrupting microtubule formation and arresting the cell cycle, in turn inducing apoptosis^[13]^. Several cancers have shown good responses to taxane treatment, however acquired resistance is common following long term exposure^[13]^. Stress hormones have been shown to mediate effects on the cell cycle in breast cancer cells, upregulating cyclin-dependent-kinase 1 (CDK-1), which lead to cell cycle progression through the G2/M stage. In this way stress hormones can reduce the efficacy of paclitaxel which acts on rapidly proliferating cells by inhibiting paclitaxel-induced G2/M arrest^[14]^. This mechanism has been further corroborated by an *in vivo* study demonstrating that the induction of psychological stress promotes tumour growth in mice treated with paclitaxel, negating its anti-tumour effect^[15]^.

**Figure 2:**
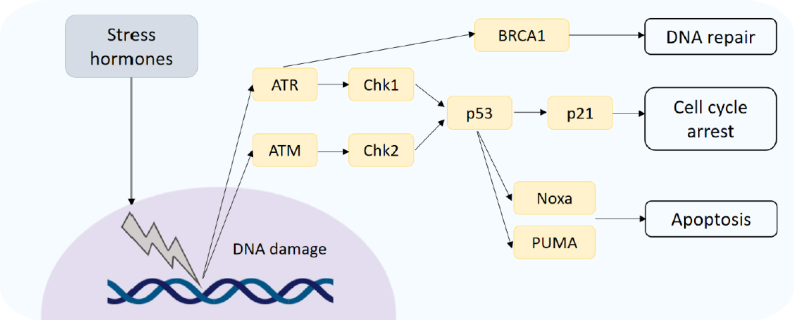
DNA damage response. DNA damage induced by stress hormones activates the DNA damage sensors ATM and ATR, which initiate downstream signal cascades controlling DNA repair, cell cycle arrest and apoptosis. ATM: ataxia-telangiectasia mutated; ATR: ataxia telangiectasia and Rad3-related

It is also proposed that the induction of DNA damage can induce resistance to chemotherapies in breast cancer. Following DNA damage, activation of the serine/threonine kinases, ATM (ataxia-telangiectasia mutated) and ATR (ataxia telangiectasia and Rad3-related), termed DNA sensors, may occur^[16]^ [Figure 2]. Furthermore, downstream substrate molecules (BRCA1 and 2, NBS, p53, and Chk2) can promote tumorigenesis. Stress hormones induce DNA damage through the production of damaging reactive oxygen and nitrogen species (ROS/RNS)^[17]^, and exposure to both GCs and catecholamines has been shown to increase levels of ROS/RNS in breast cancer cell lines^[18]^. The repair of damaged DNA has also been shown to be affected by stress hormones through interference with checkpoint kinase signalling, facilitating circumnavigation of cell cycle checkpoints which function to halt the cell and allow for repair^[19]^. In breast cancer cells treated with stress hormones the expression of DNA damage response proteins was upregulated, specifically the Kinase ATR which initiates a signalling cascade activating p21 to halt the cell cycle. This allows the cell time to repair the damaged DNA. The CDK inhibitor p21 functions to inhibit progression into the S phase of the cell cycle, and it is proposed that the arrest of cells caused by the addition of stress hormones prevents the action of chemotherapies such as paclitaxel that act on dividing cells^[15]^. The effects of GCs specifically on cell cycle arrest and the subsequent effect on cytotoxic anti-cancer treatments is reviewed extensively in^[20]^. GCs can also affect p53, indeed restraint stress decreased levels and function of p53 in mice and promoted the growth of human xenograft tumors in a p53-dependent manner^[21]^. P53 induced *P21, Noxa* and the pro-apoptotic gene *PUMA* were all decreased in response to restraint stress. The same group showed that this mechanism occurred through induction of serum- and glucocorticoid-induced protein kinase (SGK1), which in turn increases MDM2 activity and decreases p53 function potentially leading to DNA damage.

Several mechanisms have been proposed to explain the development of resistance to paclitaxel, including overexpression of multi-drug resistance (MDR) genes such as *MDR-1*, which encodes for a membrane transporter (P-glycoprotein) that can transport chemotherapeutics such as paclitaxel out of cells^[22]^. Adrenaline was shown to upregulate multi-drug resistance 1 (MDR-1) in breast cancer cells, and reduce sensitivity to paclitaxel through a reduction of accumulation of the drug within the cell. In a mouse model of psychological stress the concentrations of both cortisol and noradrenaline were increased in the plasma, and MDR-1 was significantly expressed in the primary tumour after 26 days. Tumour regression induced by doxorubicin treatment was further abolished by the induction of psychological stress, and this was reversible by knockdown of MDR-1^[23]^. These findings were corroborated in a further study using colon adenocarcinoma cells. Stimulation of the β-adrenergic receptor by adrenaline increased expression of MDR-1 and function of P-glycoprotein, and enhanced chemoresistance to 5-fluorouracil treatment^[24]^.

Administration of GCs *in vitro* have been shown to induce resistance to chemotherapies. In xenograft models of breast and ovarian cancer, pre-treatment with dexamethasone inhibited the therapeutic efficacy of paclitaxel and allowed tumour growth to continue^[25]^. It was hypothesised that GCs may prevent cells entering an apoptotic pathway after mitotic arrest, and as such interfere with chemotherapy-induced apoptotic cell death^[26]^.

However, little was understood about the mechanism by which GCs may exert anti-apoptotic effects in cancer cells challenged by chemotherapy. Activation of the GR has previously been shown promote cell survival signalling pathways and inhibit pro-apoptotic signalling in mammary epithelial cells^[27,28]^. Additional experiments revealed stimulation of the GR by dexamethasone prior to chemotherapy was able to reduce the cytotoxic potential of common chemotherapeutic agents including paclitaxel and doxorubicin in breast cancer cell lines^[29]^. It is proposed that activation of the GR induces expression of serum/glucocorticoid regulated kinase 1 (SGK-1), which protects cells from chemotherapy-induced apoptosis, and this was shown to be reversible by blockade of the GR^[30]^.

Further investigation into molecular signalling downstream of the GR revealed GCs may also contribute to cell survival through interference with mitogen-activated protein kinase (MAPK) signalling^[31]^. MAPK activation can be induced by chemotherapy agents and leads to rapid induction of apoptosis. In this study paclitaxel was shown to induce MAPK signalling and subsequent apoptosis in breast cancer cells. However, MAPK phosphatase-1 (MKP-1), an inactivator of MAPK has been shown to be transcriptionally regulated through activation of the GR. Pre-treatment with dexamethasone rapidly induced MKP-1, which was able to inhibit MAPK signalling and promote cell survival.

Drawing together these two anti-apoptotic mechanisms, and to confirm the effect of GCs on the regulation of anti-apoptotic genes, the expression of SGK-1 and MKP-1 was evaluated in a clinical setting in ovarian cancer patients^[32]^. One dose of dexamethasone was administered to patients during intra-operative biopsies, and samples of tumour collected pre and post infusion. Treatment with dexamethasone significantly increased the expression of SGK-1 and MKP-1 in ovarian tissue compared to control samples. This finding was in concurrence with the *in vitro* data obtained from ovarian cancer cell lines, which showed that expression of both SGK-1 and MKP-1 was rapidly induced by activation of the GR and could be reversed through GR blockade. Importantly this finding revealed that clinically relevant pharmacological doses of dexamethasone had significant effects on the expression of anti-apoptotic genes in tumour tissue, which may persist during chemotherapy, potentially reducing the efficacy of treatments.

**Figure 3:**
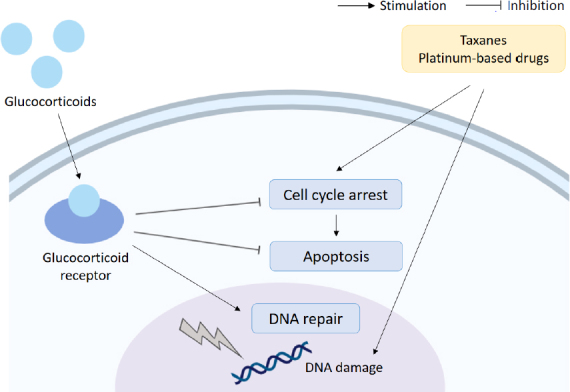
Glucocorticoids and chemoresistance. Glucocorticoids can reduce the efficacy of chemotherapeutics (taxanes and platinum-based drugs) through upregulation of cell survival signalling, downregulation of apoptosis and interference with DNA repair mechanisms

In another study of Triple Negative Breast Cancer (TNBC) resistance to paclitaxel was also induced by activation of the GR, and this was shown to occur as a result of activation of the transcription co-activator YAP. The GC mediated activation of YAP also promotes the expansion of cancer stem cells (CSCs), which are both highly metastatic and often resistant to chemotherapy^[33]^. The effects of GC on paclitaxel resistance are summarised in Figure 3.

### Platinum-based therapies

Other treatments for cancer include platinum-based chemotherapies, a class of agents used in the treatment of nearly half of all cancers. The most commonly used drugs include cisplatin, carboplatin and oxaliplatin and are used against ovarian, testicular, bladder and colon cancers amongst others^[34]^. Platinum-based therapies exert their effects through induction of apoptosis by cross-linking purine bases on DNA, interfering with DNA repair mechanisms and inducing irreparable DNA damage. However, resistance to platinum therapies is a major problem, and is thought to arise as a result of alteration to platinum transport mechanisms, DNA repair processes or apoptotic signalling^[34]^.

Stimulation of the adrenergic response has been shown to have adverse effects on the progression of ovarian cancer^[35]^. In ovarian carcinoma cells, catecholamines were able to reduce the efficacy of cisplatin and paclitaxel by inhibition apoptosis, and this effect could be modulated through inhibition of the β-adrenergic receptor^[36]^. Mechanistically, catecholamines induced the expression of DUSP1, a MAPK phosphatase linked to the promotion of chemoresistance^[37]^, through direct action of the β-2 adrenergic receptor on the promoter, which in turn protects ovarian cancer cells from apoptosis. In a xenograft model of ovarian cancer, stimulation of the adrenergic response by restraint stress significantly increased tumour burden and the expression of DUSP1. Restraint stress combined with docetaxel significantly impaired the efficacy of docetaxel and reduced docetaxel-induced apoptosis, and this could be negated by blockade of the β-AR. Knockdown of DUSP1 expression in a subsequent ovarian cancer xenograft model significantly restored the efficacy of chemotherapy - indicating that the DUSP1 expression induced by adrenergic activation has a functional role in the protection from apoptosis. The findings highlight a potential use for pharmacological inhibition of the β-AR using β-blockers to improve the efficacy of chemotherapy in the treatment of ovarian cancer patients, who report in significantly higher levels of symptoms associated with chronic stress^[38]^. β-blockers have been used successfully in the treatment of multiple cancers for many years, and more recently have been shown to correlate with increased overall survival in patients with ovarian cancer^[39]^.

Activation of the GR has also been shown to promote cell survival signalling in high grade serous ovarian carcinoma cells and inhibit carboplatin-induced cell death. Subsequent antagonism of the GR using an inhibitor was able to enhance the effect of platinum-based chemotherapy on tumour growth in a xenograft model of ovarian carcinoma^[40]^. In another model of ovarian carcinoma, the cytotoxic effects of cisplatin were also reduced by treatment with the synthetic glucocorticoid dexamethasone, which inhibited cisplatin-mediated apoptosis. It was hypothesised that dexamethasone may increase the adhesive ability of ovarian carcinoma cells, and indeed dexamethasone was shown to upregulate the expression of certain integrins which play a key role in regulating adhesion to the extracellular matrix (ECM). Inhibition of integrin signalling was able to reverse the protective effect of dexamethasone and restore the efficacy of chemotherapeutic treatment^[41]^.

Similar results with GCs have been observed in cell lines representing TNBCs. Dexamethasone was able to decrease sensitivity to cisplatin, as well induce expression of Krüppel-like factor 5 (KLF5), a transcription factor that has been shown to promotes survival and proliferation in basal breast cancers^[42]^. Furthermore, the induction of KLF5 contributed to dexamethasone mediated chemoresistance *in vivo*. Dexamethasone treatment promoted resistance to docetaxel, whereas KLF5-knockdown cells formed tumours that were sensitive to docetaxel even in the presence of dexamethasone^[42]^. Dexamethasone induced cisplatin resistance via KLF-5 to promote cell proliferation can occur via many mechanisms including through regulation of cyclin D, fibroblast growth factor binding protein 1, microsomal prostaglandin E2 synthase^[43-45]^.

In the treatment of cervical cancer, GCs may also play a role in response to therapy. Infection with certain strains of human papillomavirus (HPV) is strongly linked to an increased risk of cervical cancer. It is though that this occurs as a result of viral oncoproteins interfering with the normal functioning of the tumour suppressor p53. In HPV-positive cervical cancer cells glucocorticoids not only downregulated p53, but also a microRNA (miR-145) associated with tumour suppression and shown to be downregulated in cervical cancer tissues. The downregulation of miR-145 by cortisol correlated with an increase in resistance to the antitumor drug mitomycin C, and this was shown be a direct result of glucocorticoid-mediated expression of a viral oncoprotein^[46]^.

However, dexamethasone has also been shown to enhance the cytotoxicity of cisplatin in cervical cancer cells, through the inhibition of the transcription factor nuclear factor kappa-light-chain-enhancer of activated B cells (NF-κB)^[47]^. NF-κB is an important signal transducer that regulates certain apoptotic signalling processes, and inactivation of NF-κB can therefore promote apoptosis. In breast cancer cells a similar result was also observed, with glucocorticoid treatment inhibiting activation of NF-κB, promoting apoptosis and increasing chemosensitivity^[48]^. This directly contradicts another mechanism suggested by Fan *et al*.^[26]^ which suggests that paclitaxel promotes the release and translocation of NF-κB, which acts on apoptosis-related gene expression, promoting cell death. GCs meanwhile upregulate the expression of an inhibitor of NF-κB, which prevents this, leading to chemoresistance . These conflicting arguments therefore indicate that the effect of glucocorticoids on chemotherapy- induced apoptosis mediated by NF-κB is largely dependent on the cell type and stimulus.

Herr *et al*.^[49]^ also investigated the effects of GCs on solid cancer cell lines alongside lymphoid cells. Human lung and cervical cancer carcinoma cells were implanted as xenografts, and weekly does of cisplatin were given alongside dexamethasone added to the drinking water. It was subsequently observed that dexamethasone inhibits the efficacy of cisplatin cytotoxicity in the lung cancer xenograft model, and this was replicated *in vitro* in a human cervical cancer line. Blockade of the GR using RU486 was found to significantly reverse the GC-mediated reduction of apoptosis. The induction of apoptotic signalling by chemotherapy agents and irradiation is mediated by caspase cleavage and the cross-linking of death receptors. On a mechanistic level, treatment with dexamethasone interfered with key apoptotic molecules induced by cisplatin, downregulating caspase-8 and caspase-9, as well as death receptor ligands CD95-L (Fas ligand) and TRAIL. In a later study this result was corroborated, with dexamethasone inhibiting TRAIL-induced apoptosis in thyroid cancer cells^[50]^. The opposite effect was observed in lymphoid cells, indicating the anti-apoptotic effects of GCs are specific to solid tumours. Over-expression of caspase-8 and 9 was able to sensitise dexamethasone treated lung carcinoma cells to cisplatin *in vivo*, significantly reducing tumour growth compared to dexamethasone/cisplatin treated. Furthermore, in hepatic cells, Yang and colleagues have shown that dexamethasone can negatively affect cisplatin induced apoptosis by increasing anti apoptotic genes BCL-2 and BCL-xl^[51]^.

In a lung carcinoma cell line cell treated with cisplatin and dexamethasone, not only were the cells protected from apoptosis, they were able to recover and continue to proliferate after 3 weeks^[52]^. Dexamethasone also inhibited cisplatin-induced apoptosis and promoted cell growth in a panel of ovarian^[53]^ and pancreatic^[54]^ cancer cell lines, and similar results were observed with gemcitabine and dexamethasone co-treatment. In primary ovarian cancer cells isolated from fresh surgical samples or pleural effusions, dexamethasone prevented cell death and increased viability of cells treated with chemotherapeutics and γ-irradiation. The same effect was also observed in primary tumour cells isolated from pancreatic carcinomas. When implanted *in vivo* treatment with dexamethasone negated the therapeutic effect of cisplatin, promoting rapid growth of the pancreatic and ovarian cancer xenografts. Taken together it is likely that dexamethasone can negatively affect the efficacy of cisplatin via the GR with effects on both cell proliferation (KLF-5) and apoptotic mechanisms, e.g., elevation of anti-apoptosis genes by dexamethasone.

### Anthracyclines

Doxorubicin is a non-selective anthracycline widely used in the treatment of breast, bladder and ovarian cancer as well as types of leukaemia and lymphoma, often in combination with other therapies^[55]^. The cytotoxic effects of Doxorubicin are mediated through multiple mechanisms; the intercalation of DNA which forms doxorubicin-DNA adducts that induce cell death, and the disruption of topoisomerase-II activity, thereby preventing DNA resealing and subsequently preventing replication in rapidly dividing cells^[56]^. Furthermore, the oxidization of Doxorubicin to a semi-quinone by NAD(P)H-oxioreductases also generates free radicals increasing oxidative stress and promoting protein, lipid and DNA damage^[55,57]^. Although highly effective, and considered a mainstay of chemotherapy regimens, Doxorubicin treatment also triggers many side effects including acute cardiotoxicity^[58]^, vomiting, hair loss and inflammation^[57]^. As such it is often prescribed alongside dexamethasone to treat hypersensitivity.

Resistance to doxorubicin poses a significant problem in the treatment of aggressive breast cancers, and since there is currently no marker to predict the efficacy of Doxorubicin, it is often prescribed without a full understanding of the therapeutic gain^[59]^. Resistance to Doxorubicin has been associated with a number of factors related to its mechanisms of action, including MDR-associated proteins, alterations in DNA repair and topoisomerase activity and increased detoxification capacity^[57,60,61]^. The ability of a cell to increase protection from oxidative stress is particularly relevant in the context of Doxorubicin resistance as oxidative stress plays a role in its mechanism of action. An increase in intracellular reduced glutathione which has anti-oxidant capacities has been observed in cells with reduced Doxorubicin sensitivity^[62]^. Furthermore, studies have positively correlated expression of genes associated with increase antioxidant capacity with chemoresistance - with particular focus on nuclear factor erythroid 2-related factor (*NRF2*), a transcription factor that controls cellular redox homeostasis and detoxification by regulating several antioxidant response related genes^[63]^.

Expression of *NRF2* is upregulated in several cell lines resistant to chemotherapy drugs including cisplatin and etoposide, as well as Doxorubicin resistance breast cancer lines, and its inhibition is capable of restoring sensitivity^[63,64]^. Tamoxifen resistance was also reversed by the blockade of *NRF2* in an MCF-7 derived cell line which possessed an enhanced antioxidant expression profile^[65]^. Furthermore, the inhibition of downstream targets of *NRF2* such as heme oxygenase-1 (HO-1) have been shown to be able to decrease cell viability in leukaemia cells^[66]^. In *NRF2* knockout mice studies showed an increased susceptibility to tumour formation in response to exposure to carcinogens^[67]^, supporting the current consensus that the *NRF2* pathway functions a cell survival pathway protecting against oxidative stress that can be deregulated in tumourigenic transformation.

GCs have been shown promote cell survival signalling pathways in mammary epithelial cells^[27]^ and reduce the cytotoxic potential of common chemotherapeutic agents in breast and ovarian cancer cell lines^[29,41]^. Multiple mechanisms have been proposed to explain the pro-survival effects of GCs including increased cell adhesion and inhibition of apoptosis via the induction of anti-apoptotic proteins^[41,68]^. Dexamethasone has also been shown to modulate immunological effects in patients receiving chemotherapy, increasing the activation of regulatory T cells which are capable of suppressing anti-tumour responses^[69]^. Dexamethasone has been show to mediate conflicting effects on Doxorubicin specifically, acting as a chemosensitiser *in vivo* enhancing the inhibition of tumour growth by Doxorubicin^[48]^, and promoting chemoresistance in cancer cell lines and models^[49]^.

GCs are also known to play a role in oxidative stress in cancer. Through genomic and non-genomic pathways the GR is involved in the transactivation and transrepression of multiple anti-inflammatory responses that suppress oxidative stress^[70]^. Activation of the GR can inhibit *NRF2* activity through repression of transcription of the *NRF2* gene, and subsequently suppress the antioxidant response including the expression of antioxidant response controlled genes^[71]^. Conversely GCs have also been shown to increase levels of damaging free radicals in the context of breast cancer in a rapid non-genomic manner^[18]^. In this way, GCs may serve to increase oxidative stress which can promote decreased sensitivity to Doxorubicin through upregulation of the antioxidant response. In addition, GC-increases in oxidative stress may lead to low levels of DNA damage, decreases on DNA repair mechanisms and genome instability leading to a pro survival phenotype.

## Stress and DNA repair mechanisms

Alterations to DNA repair mechanisms are known to strongly influence the acquisition of resistance to many therapies including platinum-based therapies, due to the need for recognition and removal of platinum-DNA adducts. Activation of the DNA repair machinery slows down or halts the transition of cells through the cell cycle, and as such the ability of cells to repair the damaged DNA dictates sensitivity or resistance to platinum-based drugs^[72]^. Cortisol and noradrenaline have not only been shown to induce DNA damage, but also affect DNA repair^[19,73]^. In precancerous 3T3 cells, we have shown that stress hormones decrease UV induced DNA repair as measured by the comet assay. The addition of stress hormones increased the expression of DNA damage response proteins Chk1 and Chk2, as well as CDC25a, a member of the CDC25 family, levels of which would be expected to decrease in response to damage. Both GC and NA have been shown to reduce the rate of DNA repair in TNBC; cells treated with cortisol and assessed for DNA damage after a 20 minute repair period showed high levels of DNA damage inferring a lack of repair^[18]^. These effects may represent further possible mechanisms by which stress hormones may contribute to resistance to platinum-based therapies.

## Adrenergic stress and immune therapies

Recent animal studies have focused the attention on adrenergic stress effects on the efficiency of immune therapies. In particular, the role of stress on immune checkpoint therapy and on the immune tumour microenvironment has been shown using syngeneic models of breast cancer and melanoma. In this study, mice were exposed to mild, chronic cold stress and housed at 22 °C instead of 30 °C, causing the activation of the adrenergic signaling pathway. To block the adrenergic signaling, mice were treated with propranolol, a non-selective β-blocker. The stress effect on the immune response against cancer was assessed by evaluating the adaptive cell-mediated immune response. In particular, the presence of active CD8+ T cells was assessed in the tumour of stressed mice as well as the ratio between IFN-γ+ CD8+ and CD4+ cells. The tumour microenvironment was also studied considering the cancer-mediated immunosuppression via PD-1/PD-L1 interaction and mice were treated with a PD-1/PD-L1 inhibitor. Cold-stressed mice undergoing a treatment with propranolol showed a reduced tumour growth and an enhanced CD8+ T cell-mediated tumour immune response compared to control group housed at standard temperature. Moreover, PD-1 expression was increased in CD8+ tumour infiltrating lymphocytes (TILs) of cold-stressed mice with a decrease in the frequency in the propranolol treated group. This finding suggests that PD-1 signalling is one of the routes used by the adrenergic stress to cause immunosuppression in the tumour microenvironment and PD-1 blockade is significantly improved when cold-stressed mice are treated with propranolol^[74]^. Taken together, these results show that adrenergic stress has an effect in shaping the T cell phenotype in the context of tumour microenvironment. Also, the pharmacological blockade of the adrenergic stress signaling pathway improves to gain a pro-inflammatory effector phenotype (IFN-γ+CD8+) and reduces the anergy phenotype (PD-1high). For these reasons, a strategic treatment with a combination of propranolol and immune therapies such as PD-1/PD-L1 inhibitors has potential.

The influence of β-adrenergic signalling on T-cell targeting immune therapies was also shown in a mouse model of lymphoma. In this study, mice treated with isoprenaline, a non-selective β-adrenergic agonist, showed an acceleration in the growth of lymphoma which was reversed when the treatment was administered in mice not expressing β-adrenergic receptors (β1β2AR-KO). The effect on immune therapies was evaluated by treating mice with monoclonal antibodies against 4-1BB and PD-1 showing a significant inhibition of lymphoma growth in the 4-1BB treated group. However, when mice were also treated with isoprenaline both α4-1BB and αPD-1 immune therapies were less effective. To further investigate the effect on the cell-mediated response, mice received tumour specific CD8+ T cells with adoptive transfer prior tumour inoculation and the functional status of these cells was assessed after the isoprenaline and immune treatments. Isoprenaline significantly decreased the IFN-γ production and the cytotoxicity that T cells acquired with the α4-1BB treatment^[75]^.

**Figure 4:**
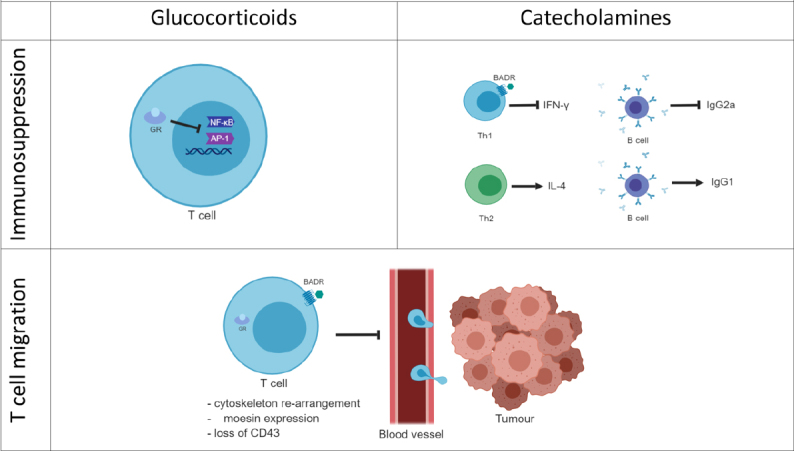
Stress hormone-mediated immune regulation. Glucocorticoids immune regulation is associated with the blockade of the pro-inflammatory gene expression. GCs bind the cytoplasmic glucocorticoid receptors (GRs) and translocate to the nucleus where they inhibit transcriptional factors such as NF-κB or AP-1 that regulate the expression of pro-inflammatory genes. Catecholamines can bind G-protein coupled adrenergic receptors on the membrane of immune T cells. The adrenergic signalling pathway blocks the production of the pro-inflammatory cytokine IFN-γ and consequently B cell production of IgG2a. On the contrary, β-adrenergic signalling is not thought to affect Th2 cells producing of IL-4 or B cells producing IgG1. Both GCs and the adrenergic hormones affect the T cell migration by regulating the T cell cytoskeleton and actin-binding proteins such as moesin. This leads to the hypothesis that stress hormones can have a role in the T cell trafficking into the tumour. Furthermore, stress hormones exposure correlates with the loss of the activation immune marker CD43.GCs: glucocorticoids; GRs: glucocorticoid receptors

Although these first studies confirm the idea that adrenergic stress weakens the efficacy of immune therapies by inhibiting the pro-inflammatory and anti-tumour response more work is necessary for a better understanding of the molecular mechanisms modulating the immune response against cancer. It is known that stress affects the cell-mediated immunity by regulating T cell subtypes and activation status. However, the literature is deficient in addressing some important aspects of the stress effect in cancer immunity that can undermine the immunotherapy potential. For instance, the efficacy of immunotherapies such as PD-1 and PD-L1 inhibitors is correlated with the immune infiltration (TILs) status of the tumour. In a study on metastatic melanoma, tumours from patients with high TILs, also known as “hot tumours”, showed a higher PD-L1 expression and a better outcome^[76]^. This consideration leads to the hypothesis that T cell infiltration into the tumour represents an indication of how successful the immunotherapy is likely to be. Since stress hormones modulate T cell migration, stress can also have a role in the T cell trafficking and infiltration into the tumour. As a final consideration, stress can affect the immune response directly or indirectly by affecting the tumour itself. In this much more complex scenario stress can create an immune hostile tumour microenvironment or regulate the tumour escape process. For these reasons, the role of stress on tumour growth, spread and angiogenesis has an important impact on how it can cooperate in undermining the tumour immune response and the efficacy of immunotherapies [Figure 4].

## Conclusion

Identification of the role of stress hormone signalling in cancer drug resistance highlights the need for greater understanding of biobehavioural factors in the treatment of cancers. Furthermore, the use of interventions including stress hormone receptor antagonism by both well-characterised and novel inhibitors may provide useful tools to complement current therapeutic strategies. Research into better anti-emetic therapies to reduce the use of synthetic glucocorticoids as co-treatments may also prove beneficial.

## Declarations

### Copyright

© The Author(s) 2019.
